# Epidemiology and survival outcomes of mucinous adenocarcinomas: A SEER population-based study

**DOI:** 10.1038/s41598-018-24540-7

**Published:** 2018-04-17

**Authors:** Guang-Dong Xie, Yi-Rong Liu, Yi-Zhou Jiang, Zhi-Ming Shao

**Affiliations:** 1 Department of Breast Surgery, Fudan University Shanghai Cancer Center; Cancer Institute, Fudan University Shanghai Cancer Center, 399 Ling-Ling Road, Shanghai, 200032 People’s Republic of China; 2 Department of Oncology, Shanghai Medical College, Fudan University, Xuhui Qu, P.R. China; 3 Institutes of Biomedical Sciences, Fudan University, Shanghai, P.R. China

## Abstract

To investigate the epidemiology, demographics and survival of mucinous adenocarcinomas (MACs), we identified 80,758 MAC patients in the Surveillance, Epidemiology and End Results (SEER) database. The reported incidence of MACs ebbed and flowed over time; however, a significant increase in reported annual age-adjusted incidences of MACs in the appendix, lung and bronchus was observed from 1981 to 2014. The demographics and outcomes of MACs differed by anatomic sites. MACs of the stomach had the largest percentage of poorly differentiated or undifferentiated tumors (41.2%), while MACs of the appendix and pancreas were associated with more advanced tumor stage (P < 0.001). MACs of the pancreas, lung and bronchus and stomach showed worse survival than other sites, despite localized, regional or distant stage (P < 0.001). In univariate and multivariate analysis, site, tumor grade, tumor stage, regional nodes, sex, race, surgery and year of diagnosis were identified as independent prognostic factors of cancer-specific survival. In conclusion, the incidence of MACs of certain specific sites, such as the appendix, lung and bronchus, is rapidly increasing. We also revealed a series of prognostic factors of MACs, including tumor sites, tumor grade and tumor stage, which may improve the current understanding of the clinical and biological patterns of MACs.

## Introduction

Mucinous adenocarcinomas (MACs) are a group of malignant tumors that originate from epithelial tissue and are characterized by abnormal mucus secretion. According to WHO classification, the diagnosis of mucinous adenocarcinoma requires that the tumor cells secrete abundant extracellular mucin involving more than 50% of the tumor volume^1^. MACs are commonly observed in colorectal cancer (approximately 10–15% of all colorectal cancers^2^) but can also occur in other glandular organs, such as the breast, stomach, ovary, and lung.

Although some studies have described the incidence, racial, sex, and primary tumor site distributions and survival durations in MAC patients from the United States and the Netherlands^3–5^, clinic-pathological factors of MACs remain largely unknown. For example, the incidence, particularly the time-trend of incidence in the general population has not been thoroughly examined. In addition, the prognosis of MACs is controversial compared with that of non-MACs. Several studies have indeed shown a worse survival in colorectal MACs^6,7^, whereas others did not identify any adverse prognostic effect^8,9^. However, for mucinous breast cancer, nearly all studies uniformly reached the same conclusion, suggesting that individuals with this cancer type have a better outcome than patients with infiltrating ductal carcinoma^10–12^. Debates have persisted regarding the relationship between the primary tumor site and cancer-specific survival in MAC cases.

To date, there have been several published population-based studies of MACs. Most MAC studies are case reports or clinical series with sample sizes at a single anatomic site. Large-scale studies investigating the prognosis of different primary MAC sites and other prognostic factors are lacking. Therefore, we conducted a population-based study using the data from the Surveillance, Epidemiology, and End Results (SEER) program to determine the epidemiology, demographics, clinical features, and survival of patients with MAC.

## Results

### Incidence

**Figure 1:**
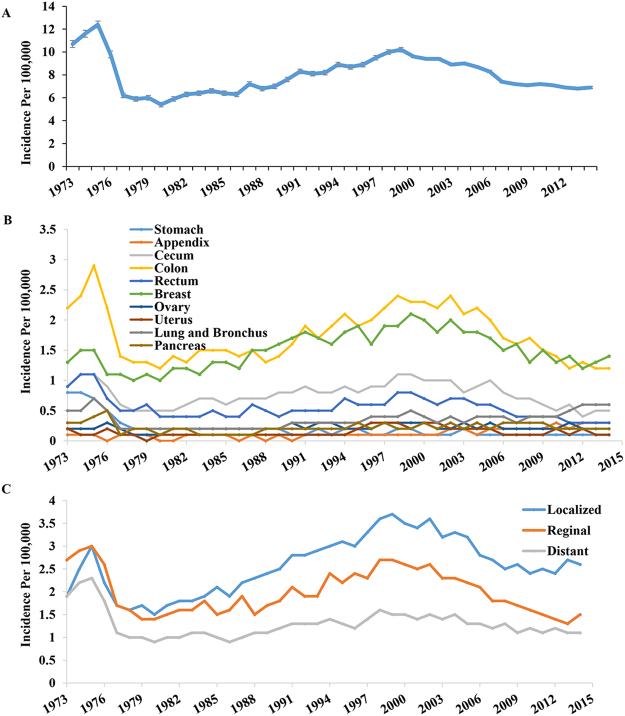
The incidence of Mucinous adenocarcinomas (MACs) over time, by site and by disease stage. (**A**) Incidence per 100,000 for Mucinous adenocarcinoma from 1973 to 2014. The reported incidence of MACs ebbed and flowed over time. (**B**) Time-trend analyses of the incidence of MACs by primary tumor site. Our study categorized 10 most common sites of MACs: stomach, appendix, cecum, colon (excluding appendix and cecum), rectum, breast, ovary, uterus, lung and bronchus, and pancreas. The incidence trends of MACs of different sites were similar to the one in (**A**) excerpt for appendix, lung and bronchus which increased significantly from 1981 to 2014 (P < 0.001). (**C**) Time-trend analyses of the incidence of MACs by disease stage at diagnosis. The incidence trends of MACs of different stages were similar to the one in (**A**).

We used the data from the SEER 9, 13, and 18 registries to calculate the annual age-adjusted incidence of MACs by year (SEER 9, 1973 to 1991; SEER 13, 1992 to 1999; and SEER 18, 2000 to 2014). We observed a fluctuation in the annual age-adjusted incidence of MACs as shown in Fig. 1A. The incidence decreased from 1973 (10.7/100,000) to 1980 (5.4/100,000), steadily increased to 10.2/100,000 in 1999, and subsequently declined from 9.6/100,000 in 2000 to 6.9/100,000 in 2014. We later performed time-trend analyses of the incidence of MACs to compare the annual percent change (APC) among the eras from 1973–1980 (APC = −12.2, P < 0.05), 1981–1999 (APC = 3.3, P < 0.05) and 2000–2014 (APC = −3.4, P < 0.05). Separate time-trend analyses by primary tumor site (Fig. 1B) and disease stage at diagnosis (Fig. 1C and B) showed the same trend over time, except for the appendix, lung and bronchus. Of these two sites, the MAC incidence significantly increased from 1981 to 2014, and their APCs also showed statistical significance from 1973 to 2014 (MACs of appendix, APC = 4.9, P < 0.05; MACs of lung and bronchus, APC = 1.2, P < 0.05). Detailed APC data in the incidence of MACs are presented in Table 1.

**Table 1:** Annual percent change of the incidence for MACs in different sites by era/SEER Database.

	1973–1980	*P*-Value	1981–1999	*P*-Value	2000–2014	*P*-Value	1973–2014	*P*-Value
Primary sites								
Stomach	−21.5	*	−2.1	*	−4.7	*	−5.2	*
Appendix	−2.9		5.4	*	6.3	*	4.9	*
Cecum	−13	*	3.3	*	−5.6	*	−0.3	
Colon^a^	−11.8	*	3.4	*	−5.4			
Rectum	−13.6	*	3.4	*	−5.9	*	−1.3	*
Breast	−5.1	*	3	*	−3.3	*	0.4	
Ovary	−8.9		3.1		−0.8		0.6	*
Uterus	−16	*	8.6	*	−5	*	1.2	
Lung and Bronchus	−16.9	*	5.1	*	4.8	*	1.2	*
Pancreas	−14.5		3.2	*	−3.3	*	−0.2	
All sites	−12.2	*	3.3	*	−3.4	*	−0.1	
Disease stage								
Localized	−7.8	*	4.5	*	−2.8	*	0.9	*
Regional	−11.7	*	3.4	*	−5.0	*	−0.3	
Distant	−13.9	*	2.7	*	−2.3	*	−0.4	

^a^Colon excluding appendix and cecum *P < 0.05.

### Demographic and clinical characteristics of MACs

The demographic and clinical characteristics of the study cohort based on MACs according to different sites are shown in Table 2. Of the 80,758 patients with MAC, 27,072 (33.5%) patients were registered in 1973–1999, and 53,686 (66.5%) patients were registered after 2000. The mean and median ages of all MAC patients were 66.3 and 68.0 years, respectively. We next divided the patients into three groups according to their age at diagnosis (<31, 31–60 and >60) and examined the relationship of age with primary tumor sites. The age at diagnosis significantly varied among different primary tumor sites (P < 0.001). Over 98.8% of patients with MAC were over 30 years old, and more than 60% of MACs were aged >60 years in the entire MAC population, except MACs of the appendix, ovary and uterus (42.6%, 41.2%, and 47.2%, respectively). These three tumor sites had lower mean (58.0, 56.5, and 59.5, respectively) and median onset ages (58.0, 56.0, and 60.0, respectively) compared with those of the other sites.

**Table 2:** Patient Characteristics according to Different Primary Tumor Sites.

	All sites	Stomach	Appendix	Cecum	Colon^a^	Rectum	Breast	Ovary	Uterus	Lung and Bronchus	Pancreas	Other sites	*P*-Value
	N = 80758(%)	N = 2237(%)	N = 2488(%)	N = 8894(%)	N = 20722(%)	N = 7054(%)	N = 18544(%)	N = 3308(%)	N = 2212(%)	N = 5086(%)	N = 3728(%)	N = 6485(%)	
Year of diagnosis													<0.001
1973–1999	27072(33.5)	1239(55.4)	351(14.1)	3065(34.5)	6978(33.7)	2648(37.5)	5879(31.7)	1042(31.5)	735(33.2)	1537(30.2)	1201(32.2)	2397(37)	
2000–2014	53686(66.5)	998(44.6)	2137(85.9)	5829(65.5)	13744(66.3)	4406(62.5)	12665(68.3)	2266(68.5)	1477(66.8)	3549(69.8)	2527(67.8)	4088(63)	
Age													<0.001
<31	962(1.2)	12(0.5)	57(2.3)	46(0.5)	251(1.2)	99(1.4)	103(0.6)	266(8)	30(1.4)	25(0.5)	9(0.2)	64(1)	
31–60	25566(31.7)	644(28.8)	1372(55.1)	2020(22.7)	5512(26.6)	2631(37.3)	5680(30.6)	1679(50.8)	1131(51.1)	1593(31.3)	1054(28.3)	2250(34.7)	
>60	54230(67.2)	1581(70.7)	1059(42.6)	6828(76.8)	14959(72.2)	4324(61.3)	12761(68.8)	1363(41.2)	1051(47.5)	3468(68.2)	2665(71.5)	4171(64.3)	
Sex													<0.001
Female	52677(65.2)	804(35.9)	1398(56.2)	5101(57.4)	11046(53.3)	2980(42.2)	18463(99.6)	3308(100)	2212(100)	2463(48.4)	1921(51.5)	2981(46)	
Male	28081(34.8)	1433(64.1)	1090(43.8)	3793(42.6)	9676(46.7)	4074(57.8)	81(0.4)	0(0)	0(0)	2623(51.6)	1807(48.5)	3504(54)	
Race													<0.001
White	66497(82.3)	1677(75)	2060(82.8)	7529(84.7)	17320(83.6)	5938(84.2)	15008(80.9)	2664(80.5)	1860(84.1)	4221(83)	3064(82.2)	5156(79.5)	
Black	7664(9.5)	270(12.1)	201(8.1)	883(9.9)	2063(10)	579(8.2)	1673(9)	286(8.6)	106(4.8)	481(9.5)	359(9.6)	763(11.8)	
Others	6315(7.8)	286(12.8)	216(8.7)	473(5.3)	1290(6.2)	527(7.5)	1768(9.5)	347(10.5)	228(10.3)	373(7.3)	300(8)	507(7.8)	
Unknown	282(0.3)	4(0.2)	11(0.4)	9(0.1)	49(0.2)	10(0.1)	95(0.5)	11(0.3)	18(0.8)	11(0.2)	5(0.1)	59(0.9)	
Grade													<0.001
I	15753(19.5)	100(4.5)	823(33.1)	828(9.3)	2039(9.8)	645(9.1)	7354(39.7)	834(25.2)	982(44.4)	1215(23.9)	414(11.1)	519(8)	
II	28961(35.9)	470(21)	686(27.6)	4778(53.7)	10631(51.3)	3344(47.4)	4453(24)	840(25.4)	667(30.2)	1150(22.6)	685(18.4)	1257(19.4)	
III and IV	12401(15.4)	922(41.2)	258(10.4)	1893(21.3)	4099(19.8)	1404(19.9)	745(4)	518(15.7)	241(10.9)	668(13.1)	441(11.8)	1212(18.7)	
Unknown	23643(29.3)	745(33.3)	721(29)	1395(15.7)	3953(19.1)	1661(23.5)	5992(32.3)	1116(33.7)	322(14.6)	2053(40.4)	2188(58.7)	3497(53.9)	
Stage													<0.001
Localized	31597(39.1)	327(14.6)	586(23.6)	2506(28.2)	5676(27.4)	1584(22.5)	15583(84.0)	1513(45.7)	1456(65.8)	1207(23.7)	290(7.8)	869(13.4)	
Regional	24605(30.5)	877(39.2)	427(17.2)	4087(46)	9344(45.1)	3710(52.6)	2194(11.8)	206(6.2)	464(21)	982(19.3)	1015(27.2)	1299(20)	
Distant	19167(23.7)	876(39.2)	1396(56.1)	2228(25.1)	5427(26.2)	1518(21.5)	425(2.3)	1476(44.6)	209(9.4)	2093(41.2)	2264(60.7)	1255(19.4)	
Unknown	5389(6.7)	157(7)	79(3.2)	73(0.8)	275(1.3)	242(3.4)	342(1.8)	113(3.4)	83(3.8)	804(15.8)	159(4.3)	3062(47.2)	
Tumor size													<0.001
≤2.0 cm	14480(17.9)	368(16.5)	479(19.3)	1556(17.5)	3641(17.6)	1218(17.3)	3437(18.5)	643(19.4)	367(16.6)	970(19.1)	699(18.8)	1102(17)	
2.1–5.0 cm	20708(25.6)	494(22.1)	669(26.9)	2316(26)	5327(25.7)	1722(24.4)	4820(26)	823(24.9)	536(24.2)	1344(26.4)	981(26.3)	1676(25.8)	
>5.0 cm	19775(24.5)	511(22.8)	669(26.9)	2152(24.2)	5059(24.4)	1706(24.2)	4617(24.9)	768(23.2)	532(24.1)	1289(25.3)	911(24.4)	1561(24.1)	
Unknown	25795(31.9)	864(38.6)	671(27)	2870(32.3)	6695(32.3)	2408(34.1)	5670(30.6)	1074(32.5)	777(35.1)	1483(29.2)	1137(30.5)	2146(33.1)	
Regional nodes positive													<0.001
None	31273(38.7)	220(9.8)	1018(40.9)	3631(40.8)	8223(39.7)	1923(27.3)	11825(63.8)	1238(37.4)	799(36.1)	1220(24)	381(10.2)	795(12.3)	
Yes	18290(22.6)	587(26.2)	327(13.1)	3625(40.8)	7646(36.9)	2578(36.5)	1490(8)	145(4.4)	205(9.3)	631(12.4)	460(12.3)	596(9.2)	
Unknown	31195(38.6)	1430(63.9)	1143(45.9)	1638(18.4)	4853(23.4)	2553(36.2)	5229(28.2)	1925(58.2)	1208(54.6)	3235(63.6)	2887(77.4)	5094(78.6)	
Surgery													<0.001
No	9080(11.2)	442(19.8)	236(9.5)	223(2.5)	1083(5.2)	615(8.7)	734(4)	313(9.5)	203(9.2)	2126(41.8)	2049(55)	1056(16.3)	
Yes	48104(59.6)	637(28.5)	1967(79.1)	6034(67.8)	13518(65.2)	4130(58.5)	12783(68.9)	2080(62.9)	1422(64.3)	1636(32.2)	603(16.2)	3294(50.8)	
Unknown	23574(29.2)	1158(51.8)	285(11.5)	2637(29.6)	6121(29.5)	2309(32.7)	5027(27.1)	915(27.7)	587(26.5)	1324(26)	1076(28.9)	2135(32.9)	

^a^colon excluding appendix and cecum.

The MACs cohort in the present study included 65.2% female patients. Lung and bronchus, stomach and rectum had a higher proportion of male cases (P < 0.001). In addition, 82.3% of cases were white, far more than black people (9.5%) and other races (mainly Asians, 7.8%). Notably, MACs of the stomach had a relatively lower percentage of white patients (only 75%) than those of other sites (P < 0.001).

Tumor size and regional nodes were also examined under different sites, and we observed that MACs of the colon and rectum were more likely to have regional-positive nodes. Surgery is one of the most important therapies for MACs, but the present results showed that surgery rates significantly varied across different sites, with tumors of the stomach, lung and bronchus and pancreas showing significantly lower surgery rates (28.5%, 32.2%, and 16.2%, respectively) than any other site (P < 0.001). According to the present study, the MACs in these sites were more likely to present high tumor grades or metastasis, and this finding may partly explain why the surgery rates of these cancers were lower than those of other tumors (Table 2).

Table 2 also shows the distribution of MACs tumor grade and tumor stage by primary sites. The information on tumor grade and tumor stage was available for 70.7% and 93.3% of the patients, respectively. Over 39.7% of MACs in the breast and 44.4% of MACs in the uterus were well-differentiated, which is greater than the distribution in any other site (P < 0.001). Tumors of the stomach presented a typically high grade, with the highest proportion (41.2%) being poorly differentiated (G3) or undifferentiated (G4) tumors cases. Similarly, the majority of breast and uterus MACs were localized (84.0% and 65.8%, respectively) compared to other sites, whereas tumors in the stomach, appendix, ovary, pancreas, lung and bronchus had a relatively higher proportion of distant stage.

### Survival analysis

**Figure 2:**
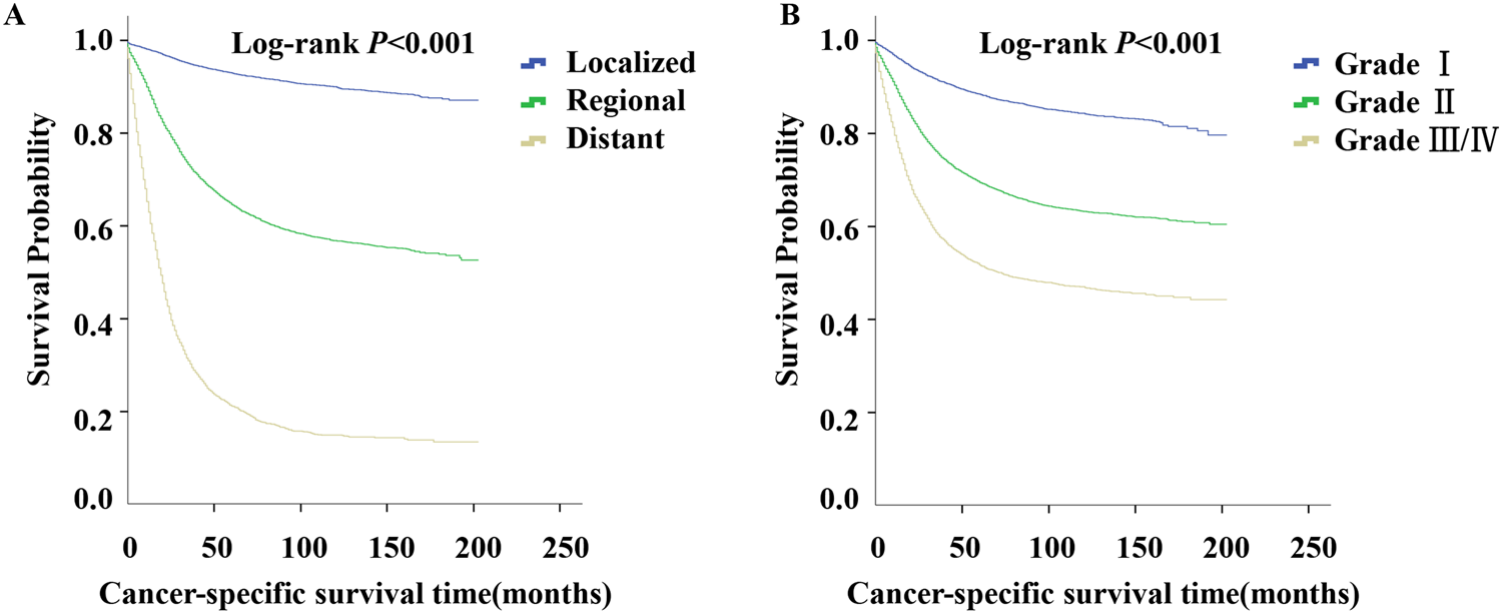
Survival analysis of MACs by tumor stage and tumor grade. (**A**) Cancer-specific survival analysis of MACs by tumor stage. As expected, localized MACs had favorable survival than regional and distant disease (log-rank P < 0.001). (**B**) Cancer-specific survival analysis of MACs tumor grade. The strong prognostic impact of histological grade for MACs was observed (log-rank P < 0.001).

We performed survival analysis in MACs by tumor stage and tumor grade. The 5-year survival rate in patients with localized, regional and distant MACs were 92.9% (95% CI: 92.5–93.4%), 64.5% (95% CI: 63.6–65.4%), and 21.0% (95% CI: 19.8–22.3%), respectively. Kaplan-Meier disease-specific survival analysis showed that localized MACs had a more favorable survival than regional and distant diseases (Fig. 2A, log-rank P < 0.001). Additionally, patients with G3 and G4 tumors had identical survival curves (log-rank P = 0.088), and the difference in survival duration among the patients with well differentiated (G1), moderately differentiated (G2), and G3/G4 MACs was statistically significant (Fig. 2B, log-rank P < 0.001).

**Figure 3:**
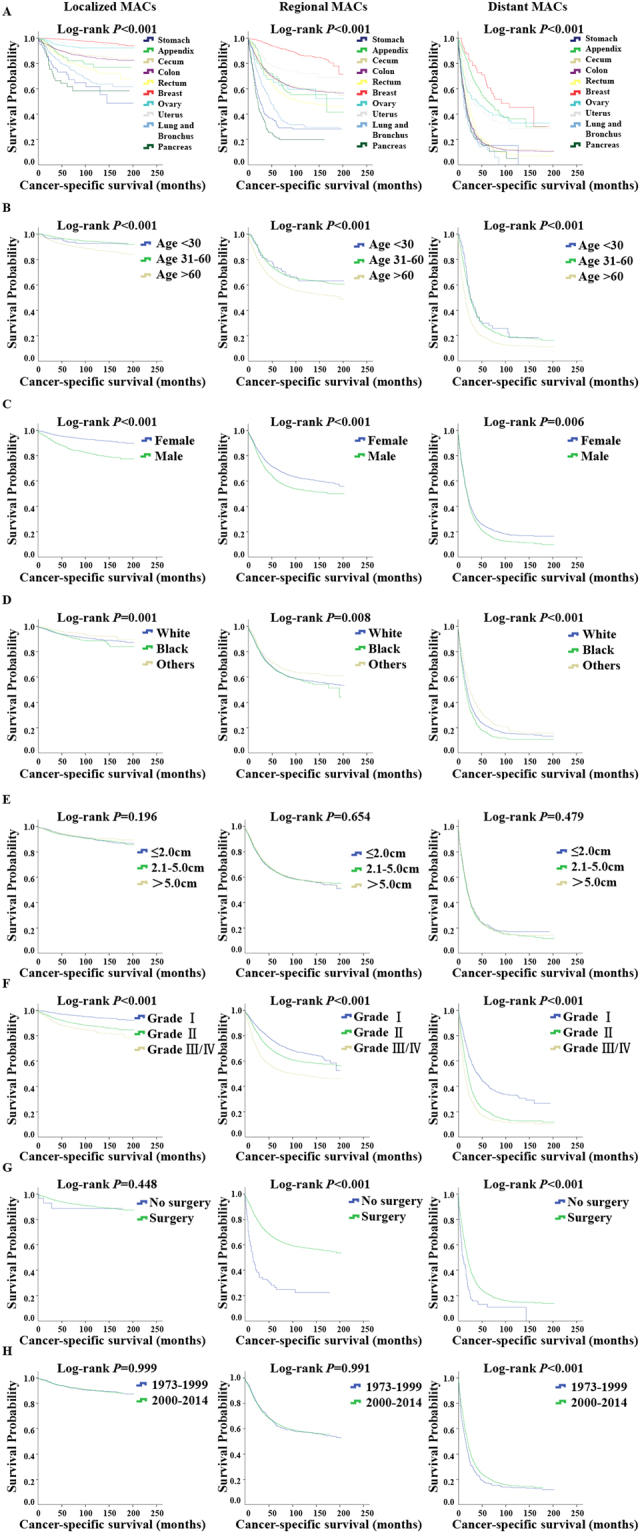
Survival analysis by primary tumor site, age, sex, race, tumor size, lymph node, tumor grade, surgery and year of diagnosis for MACs in different stages. (**A**–**H**) Left, localized MACs; Middle, regional MACs; Right, distant MACs. (**A**) Cancer-specific survival analysis by primary tumor site. MACs of the pancreas, lung and bronchus and stomach were associated with worse survival compared to MAC of any other sites, while MACs of breast had the best survival outcomes in localized, regional and distant diseases (log-rank P < 0.001). (**B**) Cancer-specific survival analysis by age. Younger patients had better survival than older patients (log-rank P < 0.001). (**C**) Cancer-specific survival analysis by sex. Male patients with MAC appeared to have worse survival durations than female patients whether in localized, regional or distant disease stage (log-rank P < 0.001). (**D**) Cancer-specific survival analysis by race. White patients presented better CSS than black people, but significantly worse than other races (mainly Asians) in all disease stage (log-rank P < 0.05). (**E**) Cancer-specific survival analysis by tumor size. No significant difference was observed in CSS among MACs with different tumor size no matter in localized, regional or distant disease (P = 0.196, P = 0.654, P = 0.479, respectively). (**F**) Cancer-specific survival analysis by tumor grade. MACs with higher grade presented increased risk of death compared to lower grade, which were consistent with the result shown in Fig. 2B. (**G**) Cancer-specific survival analysis by surgery. Surgery was associated with better outcomes in regional and distant MACs (log-rank P < 0.001). (**H**) Cancer-specific survival analysis by year of diagnosis. A remarkable improvement in survival of the 2000–2014 cohort compared to 1973–1999 cohort was seen in distant disease stage (log-rank P < 0.001).

Since MACs stage was such an important predictor of prognosis, we stratified tumors using the SEER Historic Stage A system (localized, regional, and distant stage) for further survival analysis. We next examined the potential prognostic factors for survival by disease stage and identified the primary tumor site as a powerful predictor of survival duration (Fig. 3A, log-rank P < 0.001). Among patients with localized disease at diagnosis, the 5-year survival rate ranged from 27.1% (95% CI: 22.5–31.7%) for patients with MACs of the pancreas to 97.7% (95% CI: 97.4–98.0%) for MACs of the breast (log-rank P < 0.001). In addition, the five-year survival rate among patients with regional MACs varied from approximately 11.3% (pancreas tumors, 95% CI: 9.0–13.6%) to greater than 92.8% (breast tumors, 95% CI: 91.1–94.5%). While among patients who presented with metastasis, MACs of lung and bronchus had worse survival compared to MACs of the pancreas (median survival durations: 12.0 vs. 16.0 months, log-rank P < 0.001), whereas MACs of the breast still had the best survival, exhibiting a median survival time of 91.0 months (95% CI: 42.9–139.1 months). In the Kaplan-Meier disease-specific survival analysis of patients with localized, regional or distant disease, the MACs of the pancreas, lung and bronchus and stomach were consistently associated with worse survival compared to the MACs of any other sites (log-rank P < 0.001). As previously described, these three sites of MACs also had relatively higher tumor grade or metastasis rates and lower surgery rates among different primary sites. Taken together, these facts may indicate that the MACs of the pancreas, lung and bronchus and stomach had more aggressive biological behavior and poorer prognosis than the MACs of other sites, regardless of disease stage.

Other factors, including age at diagnosis, sex, race, tumor grade, surgery and year of diagnosis were also prognostic factors of survival. We further explored the effect of each factor on survival at different tumor stages. The results showed that younger patients had better survival than older patients, regardless of tumor stage (Fig. 3B, log-rank P < 0.001). Female patients with MAC consistently exhibited better survival durations than male patients, despite localized, regional or distant disease stage (Fig. 3C, log-rank P < 0.001). We also analyzed the effect of races on the survival of MACs and observed that white patients presented better cancer-specific survival (CSS) than black patients but significantly worse survival than other races (mainly Asians) in all disease stages (Fig. 3D, log-rank P < 0.001). Using the SEER database, we categorized patients into three groups according to tumor size at diagnosis (≤2.0 cm, 2.1–5.0 cm and >5.0 cm). However, no significant difference was observed in CSS among MACs with different tumor size, despite localized, regional or distant disease (P = 0.196, P = 0.654, P = 0.479, respectively).

In addition, we conducted survival analyses in patients with G1, G2, and G3/G4 MACs stratified by disease stage. The survival curves showed that histological grade was a strong predictor of prognosis in all disease stages (Fig. 3F, log-rank P < 0.001), which is consistent with the results shown in Fig. 2B. To determine the relationship between surgery and the prognosis of MAC patients in different disease stages at diagnosis, we directly compared the survival durations in the operative group with those in the non-operative group and observed that surgery was associated with better outcomes in regional and distant MACs (Fig. 3G, log-rank P < 0.001). We also examined whether the treatment of MACs improved over time. Thus, we separated the patients into two cohorts (1973–1999 and 2000–2014) and observed a remarkable improvement in survival in the 2000–2014 cohort compared to that in the 1973–1999 cohort in distant stage (Fig. 3H, log-rank P < 0.001).

Univariate and multivariate Cox regression analysis was conducted to identify factors associated with cancer-specific mortality, and HRs and 95% CIs were reported. Primary tumor site, tumor grade, tumor stage, regional nodes, sex, race, surgery and year of diagnosis were identified as independent prognostic factors in the multivariate Cox model after adjusting for other confounding factors (Table 3).

**Table 3:** Univariate and Multivariate Cox Proportional Hazards Analysis of Cancer-specific Survival (CSS) of MACs.

	Univariate analysis		Multivariate analysis	
	HR (95% CI)	*P*-value	HR (95% CI)	*P*-Value
Year of diagnosis				
1988–1999	Reference		Reference	
2000–2014	0.89(0.83–0.96)	0.003	0.93(0.86–1.00)	0.050
Age				
<31	Reference		Reference	
31–60	0.94(0.78–1.13)	0.510	1.08(0.90–1.30)	0.418
>61	1.21(1.00–1.45)	0.046	1.65(1.37–1.98)	<0.001
Sex				
Female	Reference		Reference	
Male	2.01(1.93–2.09)	<0.001	1.07(1.03–1.12)	0.001
Race				
White	Reference		Reference	
Black	1.18(1.11–1.26)	<0.001	1.14(1.07–1.22)	<0.001
Others	0.81(0.75–0.87)	<0.001	0.86(0.79–0.93)	<0.001
Primary site				
Stomach	Reference		Reference	
Appendix	0.40(0.34–0.47)	<0.001	0.51(0.43–0.60)	<0.001
Cecum	0.47(0.41–0.53)	<0.001	0.59(0.53–0.67)	<0.001
Colon^a^	0.45(0.40–0.50)	<0.001	0.62(0.55–0.70)	<0.001
Rectum	0.52(0.46–0.58)	<0.001	0.70(0.61–0.79)	<0.001
Breast	0.03(0.03–0.04)	<0.001	0.13(0.11–0.15)	<0.001
Ovary	0.24(0.20–0.28)	<0.001	0.56(0.47–0.68)	<0.001
Uterus	0.15(0.12–0.19)	<0.001	0.37(0.30–0.46)	<0.001
Lung and Bronchus	0.52(0.45–0.60)	<0.001	1.22(1.05–1.41)	0.011
Pancreas	1.21(1.04–1.42)	0.016	1.53(1.30–1.79)	<0.001
Other sites	0.80(0.69–0.93)	0.003	1.00(0.86–1.16)	0.965
Grade				
I	Reference		Reference	
II	2.77(2.6–2.95)	<0.001	1.23(1.15–1.32)	<0.001
III and IV	5.03(4.7–5.39)	<0.001	1.57(1.46–1.69)	<0.001
Stage				
Localized	Reference		Reference	
Regional	5.47(5.13–5.82)	<0.001	1.83(1.69–1.98)	<0.001
Distant	19.81(18.55–21.15)	<0.001	6.87(6.33–7.45)	<0.001
Tumor size				
≤2.0 cm	Reference		Reference	
2.1–5.0 cm	1.04(0.98–1.09)	0.188	1.03(0.97–1.08)	0.352
>5.0 cm	1.00(0.94–1.06)	0.965	0.98(0.92–1.04)	0.475
Regional nodes positive				
None	Reference		Reference	
Yes	5.24(5.02–5.48)	<0.001	2.16(2.04–2.28)	<0.001
Surgery				
No	Reference		Reference	
Yes	0.23(0.20–0.26)	<0.001	0.63(0.54–0.73)	<0.001

Abbreviations: HR, hazard ratio; CI, confidence interval. ^a^Colon excluding appendix and cecum.

## Discussion

To the best of our knowledge, this study is the first to investigate the incidence and prognostic factors for MAC patients across different primary tumor sites based on the SEER database. In this retrospective study, we observed a fluctuation in the annual age-adjusted incidence of MACs during 1973 to 2014. However, the explanation behind this change remained poorly understood and should be further explored. We also observed that separate time-trend analyses differed by primary tumor site and the reported incidence of MACs of the appendix, lung and bronchus significantly increased from 1981 to 2014. Whether changes in dietary habits, environmental factors, and use of certain medications resulted in increased reported incidence of MACs of various types was unknown. However, this increase was likely caused in part by improvements in the pathological classification of these tumors. Additionally, the widespread use of technology for cancer screening likely contributed to the increase in the reported incidence of lung MACs^13^.

MACs can occur at different anatomic sites, but whether these tumors share common clinical features or survival durations remains unknown. The present study suggested that disease stage and grade, age, sex, race, and period of diagnosis are associated with primary tumor site. In particular, MACs of the stomach, appendix, ovary, pancreas, lung and bronchus were associated with more advanced tumor stage, and MACs of the stomach had the largest percentage of G3/G4 cases (41.2%). Furthermore, disease-specific survival analyses also revealed that the survival of MACs of the pancreas, lung and bronchus and stomach were significantly worse than that of the other sites, indicating that primary tumor site is an important prognostic factor for MACs. MACs of the pancreas, lung and bronchus and stomach were also associated with a lower surgery rate, the reason for which was perhaps in part due to their aggressive biological behavior. These findings indicate distinct patterns of epidemiology and survival outcomes, even for the same pathological type of MACs at different sites. Similar results were observed for other cancer pathological types, such as neuroendocrine tumors^14^ or adenoid cystic carcinomas^15^. These results suggested that for the MACs of certain specific sites, early detection and aggressive treatment should be administered to improve the prognosis. Further studies are needed to compare the intrinsic differences of MACs at different sites at the molecular and genetic levels.

Another finding of this study was other prognostic factors associated with the survival of MACs in addition to primary tumor site, including disease stage, tumor grade, regional nodes, sex, race, surgery and year of diagnosis, as previously reported^10^. Univariate and multivariate analysis using the Cox proportional hazards model showed that these parameters were independent prognostic factors after adjusting for other clinicopathological factors. Moreover, most factors, except surgery and year of diagnosis, had prognostic significance, despite localized, regional or distant disease stage. This result is somewhat different from that of a previous study, investigating the impact of tumor grade on survival, stratified by tumor stage, in mucinous appendiceal carcinoma^16^. This study included a total of 4491 appendiceal adenocarcinomas identified in the SEER database from 1988–2011 and concluded that in Stage III and IV mucinous tumors, higher tumor grade was significantly correlated with worse survival. However, tumor grade had no impact on CSS in stage I and II tumors. The reason for this discrepancy may be that the present study included more MAC cases, not only mucinous appendiceal carcinomas, compared to the previous study, thus the present results achieved statistical significance even in low stages. Additionally, the 7^th^ AJCC staging system for stage IV appendiceal adenocarcinomas incorporates histological grade into the staging and classifies mucinous tumors into low grade (well-differentiated) and high grade (moderate and poorly differentiated)^17^. The present study also found that distant diseases with poorly differentiated grade were associated with worse survival compared with moderately differentiated diseases in the MACs of all sites or the appendix alone (log-rank P < 0.001), which partly confirmed the conclusions of previous studies^18–20^. The evidence from the present study suggested that these two histological categories may be not sufficient, and three categories (well, moderate, and poor) would be more appropriate for mucinous appendiceal adenocarcinomas.

In the present study, we observed a statistically significant increase in survival duration among patients with distant MACs over time. One possible explanation is the development of systemic chemotherapy and targeted therapy. For example, human epidermal growth factor receptor 2(HER2) targeted therapies, including trastuzumab, pertuzumab, lapatinib and trastuzumab emtansine (T-DM1), for the treatment of metastatic breast cancer, ovarian cancer and gastric cancer greatly prolonged the survival of HER2-positive patients^21–23^. Currently, an increasing number of novel therapeutic approaches for MACs, such as immunotherapy and targeted agents, are under development. These new therapies may further improve the prognosis of patients with MAC. In addition to the changing in treatment, new diagnostic methods, earlier detection of the disease and improvement of rehabilitation quality may also contributed to the improved survival outcomes.

Several limitations in the present study should be mentioned. First, the record of tumor size and positive lymph nodes prior to 1988 were missing in the SEER database, which may decrease the sample size and power in the present multivariate analysis. Second, the information on systemic treatments, such as chemotherapy or targeted therapy, and other therapies, such as endocrine therapy for breast MACs, were unknown or incomplete. Therefore, these prognostic factors could not be obtained and adjusted for the observed results, thereby causing bias in the analysis. For example, these missing potential prognostic factors were not included in the Cox regression analysis, which perhaps decreased the ability of Cox model of adjusting the impact of other co-factors. Despite these limitations, the large sample size of nearly 80,758 patients and the ability to perform subgroup survival analysis to identify prognostic variables were important advantages of the SEER registry.

In conclusion, the results of the present study depicted the incidence of MACs in the general population and in different anatomic sites. Notably, the incidence of the MACs of several specific sites, such as the appendix and lung and bronchus, are rapidly increasing. The demographics, clinical features and survival outcomes of MACs differed by primary site. The MACs of pancreas, lung and bronchus and stomach have a worse survival, while the MACs of the breast consistently showed the best survival compared with other sites. Tailored treatment should be administered for the MACs of different anatomic sites. In addition to primary tumor site, other factors, including older age, male gender, black race, and higher tumor grade and tumor stage, were also associated with the worse outcome of MACs.

## Methods

### Patient selection

The SEER program databases contain information on cancer incidence and survival from specific geographic areas across the United States. The SEER 9, 13, and 18 registries cover approximately 9.5%, 13.8%, and 28%, respectively, of the total US population. In the present study, we obtained and analyzed the SEER data based on the November 2016 submission. Because the SEER 9, 13, and 18 registries are linked to different population datasets, we computed the age-adjusted incidence for three time periods: SEER 9, 1973 to 1991; SEER 13, 1992 to 1999; and SEER 18, 2000 to 2014. On the basis of the ICD-O-3 codes provided in the SEER database, we identified all eligible patients diagnosed with MACs (ICD-O-3 code 8480/3 for mucinous adenocarcinoma) across different primary sites. A total of 80,967 cases of MACs during 1973 to 2014 diagnosed with first and only cancer diagnosis were identified. We excluded patients diagnosed by either autopsy or death certificate. In total, 80,758 patients were eventually eligible for inclusion. Because a unified staging system for MACs is lacking, the SEER staging system was used for analysis. Tumors were classified as localized, regional, or distant. A localized MAC was defined as malignancy limited entirely to the organ of origin. A regional MAC was defined as a neoplasm tumor extension invaded beyond the limits of the organ of origin but no distant disease or involved regional lymph nodes, and a distant MAC was defined as a neoplasm that spread to areas of the body remote from the primary tumor^24^.

### Statistical analysis

Differences in demographics and clinical features were assessed with chi-square test or t-test. Cancer-specific survival was used as the primary study outcome and was calculated from the date of diagnosis to the date of death due to a specific MAC. Patients who were alive at last follow-up or died from other causes were censored. Cancer-specific survival curves were generated using the Kaplan-Meier method, and differences between curves were analyzed by log-rank tests. Univariate and multivariate Cox proportional hazard (PH) models were applied to identify factors that were associated with cancer-specific survival, and hazard ratios (HR) were reported as point estimates with 95% confidence intervals (CI). Since the record of tumor size and positive lymph nodes prior to 1988 were missing in the SEER database, we excluded MAC cases before 1988 in our Cox regression analysis. What’s more, we excluded MAC patients with unknown information of every potential prognostic factors to increase the credibility of the Cox model. A graphical approach called log-log survival curve was used to check the PH assumption over the variables included in the Cox model. These curves were approximately parallel and no evidence was found to strongly indicate the PH assumption was violated for all the variables. The incidence and trends of MACs were calculated using the rate session of SEER*Stat 8.3.4. All other statistical calculations were performed using SPSS, version 22.0 (IBM Corp, Armonk, NY). Comparative differences were considered to be statistically significant when P was less than 0.05.
